# A developmental study of eye movements in Hebrew word reading: the effects of word familiarity, word length, and reading proficiency

**DOI:** 10.3389/fpsyg.2023.1052755

**Published:** 2023-07-07

**Authors:** Hend Lahoud, David L. Share, Adi Shechter

**Affiliations:** ^1^Department of Learning Disabilities, Faculty of Education, University of Haifa, Haifa, Israel; ^2^Edmond J. Safra Brain Research Center for the Study of Learning Disabilities, University of Haifa, Haifa, Israel

**Keywords:** eye movement, Hebrew, word recognition, lexicality, reading, word length, word familiarity

## Abstract

Previous studies examining the link between visual word recognition and eye movements have shown that eye movements reflect the time-course of cognitive processes involved in reading. Whereas most studies have been undertaken in Western European languages written in the Roman alphabet, the present developmental study investigates a non-European language—Hebrew, which is written in a non-alphabetic (abjadic) script. We compared the eye-movements of children in Grades 4 to 6 (*N* = 30) and university students (*N* = 30) reading familiar real words and unfamiliar (pseudo)words of 3 letters and 5 letters in length. Using linear mixed models, we focused on the effects of word familiarity, word length, and age group. Our results highlight both universal aspects of word reading (developmental and familiarity (lexicality) effects) as well as language-specific word length effect which appears to be related to the unique morphological and orthographic features of the Semitic abjad.

## Introduction

One of the hallmarks of skilled reading is the remarkable speed and apparent effortlessness of word recognition. Most reading researchers agree that fast, efficient recognition of printed words (often termed *automatic* or *fluent* word reading) is crucial to successful reading development because it enables the reader to devote limited processing resources to comprehension (LaBerge and Samuels, 1974; Perfetti, 1985).

Although a variety of word recognition models have been proposed to explain how visual and linguistic processes interweave to allow efficient word reading (e.g., McClelland and Rumelhart, 1981; Seidenberg and McClelland, 1989; Grainger and Jacobs, 1996; Davis, 2010) the dual route model arguably remains the most influential model of word reading (Coltheart et al., 1993; Zorzi, 2010; Grainger and Ziegler, 2011; Ellis and Young, 2013). The central axiom of the Coltheart/Baron version of the dual route model (see Coltheart, 1978; Baron, 1979) is that no single procedure yields correct pronunciations of both nonwords (e.g., *slint*) and exception words (e.g., *pint*). Nonwords can only be correctly pronounced via grapheme–phoneme correspondence rules (the “non-lexical” or “sub-lexical” route); exception words require an additional procedure (the “lexical” route) because they cannot be pronounced by the rules. But if a writing system contains few if any exception words, is a second route necessary? Most alphabetic orthographies (both European and non-European) are highly regular in terms of print-to-sound relations (Daniels and Bright, 1996; Seymour et al., 2003), thus, a single rule-based mechanism should be adequate for pronouncing all (or almost all) letter strings. Consequently, a number of reading researchers have begun to question the generalizability of the dual-route architecture beyond English (e.g., Bishop and Snowling, 2004; Hutzler and Wimmer, 2004; Ziegler and Goswami, 2005).

Share (2008) proposed a non-Anglocentric variant of the dual route model designed to apply to *all* words in *all* possible orthographies, alphabetic and non-alphabetic, regardless of their degree of “regularity.” He argued that, on the one hand, *all* words are visually unfamiliar at some point in reading development; thus, the developing reader must possess a means for *independently* identifying (decoding) words encountered for the first time (Jorm and Share, 1983; Share, 1995). On the other hand, the skilled reader-to-be must eventually be able to achieve a high degree of automatization in word recognition—rapid and effortless recognition of familiar words and morphemes (LaBerge and Samuels, 1974; Perfetti, 1985; Logan, 1988, 1997) perceived as whole units via a direct-retrieval mechanism. In developmental terms, this universal “unfamiliar-to-familiar” or “novice-to-expert” dualism implies a developmental shift from slower, sequential letter-by-letter (sub-lexical) decoding to a faster, essentially parallel, whole-word or whole morpheme (lexical) procedure. This universal “unfamiliar-to-familiar” dualism serves as the theoretical framework for the present study.

Turning to the abundant empirical evidence on visual word recognition, this work has traditionally relied on behavioral measures of reading speed and accuracy but today employs a host of electrophysiological and imaging techniques. Eye-tracking of online reading is another increasingly popular tool partly because it offers a more “dynamic,” temporally sensitive approach to studying word recognition processes, and also because eye movement research has successfully demonstrated a direct link between the cognitive processes involved in word recognition and fixation durations (Rayner, 1998). Most of this work, primarily undertaken in English, has shown that word recognition takes an average of 250 ms per word among skilled readers (Rayner, 2009) and is also modulated by linguistic factors such as word length and word frequency (see Rayner, 1998 for a review). Longer words elicit longer fixation durations, and this effect has been observed not only in English (e.g., Rayner and McConkie, 1976; Rayner et al., 1996, 2011) but also in Semitic languages (e.g., Hebrew: Deutsch and Rayner, 1999; and Arabic: Paterson et al., 2015). Fixations on highly frequent words are shorter than on less frequent words (Rayner, 1977; Just and Carpenter, 1980; Inhoff, 1984; Inhoff and Rayner, 1986; Rayner and Duffy, 1986; Raney and Rayner, 1995; Rayner, 1998). Eye movement studies have also shown a “lexicality” effect which is the difference between reading real words and pseudowords. Pseudowords elicit longer fixation durations and more fixation counts as compared to real words (e.g., De Luca et al., 2002; Hautala et al., 2011). Moreover, the lexicality effect has been observed as early as the first fixation as well as in later stages of reading as seen in go-past times, regressions and re-reading of words (Wochna and Juhasz, 2013).

With regard to developmental aspects of word reading, studies using eye-tracking have also revealed that eye movement measures are affected by the reader’s proficiency, with higher proficiency associated with shorter fixation durations and longer progressive saccades. Moreover, studies examining the early stages of reading acquisition have revealed several differences in the eye movement behavior of children and skilled readers. First, the number of children’s fixations per word (“fixation count”) is higher than adults’; children tend to make two or three fixations on a single word compared to a single fixation by adults. Second, the average fixation durations for children are longer as compared to adults (over 350 ms in first grade). Third, children make more regressive eye-movements (30% of total fixations) as compared to 10% in adults (Rayner et al., 2006). Lastly, the perceptual span—the region in which information can be processed during a single fixation, is smaller for children (Rayner, 1986; Kwon et al., 2007; Häikiö et al., 2009).

Observing these differences in eye movement between children and adults, Hautala et al. (2011) suggested that the decrease in the number of fixations on a word among skilled readers reflects the shift from serial to parallel processing (as discussed above), while the higher fixation count among children and dyslexic readers reflects sub-lexical processing. By comparing adults and dysfluent children reading real and pseudowords in Finnish, Hautala et al. (2011) found that word type and word length had significant effects on fixation counts among both children and adults. However, children showed a larger word length effect compared to adults, indicating slower, more sequential letter-by-letter reading. Adults, on the other hand, showed a reduced word length effect indicating more whole-word (lexical) reading. Another study by Tiffin-Richards and Schroeder (2015) compared eye-movements of German-speaking children and adults and showed effects of word length and word frequency in reading in both groups but, once again, these effects were more salient in children. In addition, an interaction between word length and frequency, limited only to the children, revealed that the word length effect was greater for infrequent words than for frequent words. Similar to Hautala et al. (2011), Tiffin-Richards and Schroeder (2015) interpreted their results as reflecting the sequential processing (the sub-lexical procedure) characterizing children’s reading whereby longer words require more processing and thus longer fixation durations.

It is worth noting that similar eye-movement behavior is observed in young children and dyslexic readers, indicating that these patterns are associated with the reader’s proficiency rather than the age group. For instance, previous studies showed that the fixation durations of dyslexic readers are longer than for typically developing readers. In addition, dyslexic readers rarely skip words and thus their reading is characterized by a higher fixation count on words (De Luca et al., 1999, 2002; Hutzler and Wimmer, 2004; Hawelka et al., 2010; Krieber et al., 2016). Moreover, there is evidence that dyslexic readers read real words in the same way that skilled readers read pseudowords, relying on the slower sub-lexical route (De Luca et al., 2002).

Collectively, these studies permit the following conclusions: (a) there exists a direct link between lexical, sub-lexical processing and eye movement during word reading, and (b) the rapid lexical processing of familiar words by skilled readers is observed in a decrease in fixation count and in shorter fixation durations while children’s reading is characterized by a higher fixation count and longer fixation durations. Despite the impressive convergence of developmental findings regarding word reading processes and eye movements, this field of research has been almost entirely limited to studies in English and a handful of Western European languages, all written in the Roman alphabet. The study of reading is now beginning to acknowledge the variation between different languages and writing systems and the importance of exploring the full spectrum of the world’s languages and writing systems in order to determine which aspects of reading are truly universal and which are specific to the particular language family and writing system being read (Share, 2008, 2021; Frost, 2012; Daniels and Share, 2018; Smith et al., 2021; Siegelman et al., 2022).

The present developmental study examines the effects reviewed above in Hebrew – a non-European language written in a non-alphabetic writing system. Hebrew, like Arabic, is a Semitic language written in an abjad (or consonantal) writing system (read from right to left). All 22 letters represent consonants, with vowels represented (i) by four non-optional dual-purpose letters that double as vowels and (ii) a set of 13 optional diacritic-like vowel signs, primarily sub-lineal, which are the standard form of Hebrew for beginning readers, children’s literature, poetry, and liturgical texts. When fully “vowelized” with the vowel diacritics, Hebrew text is referred to as *pointed*: when the optional vowel diacritics are omitted, this form of Hebrew is referred to as *unpointed* which is the standard form for skilled readers (Share, 2017). It is also important to point out that Hebrew has a rich and complex non-concatenative derivational and inflectional morphology. Additionally, function words can appear either as free or as bound morphemes that are affixed to content words.

Very few studies of eye movements in Hebrew reading have been undertaken – all on skilled adult readers. Nonetheless, this work has confirmed the findings from European languages demonstrating the influence of word length (Deutsch and Rayner, 1999) and word frequency (Mor and Prior, 2021). However, no study, to our knowledge, has examined these effects in Hebrew from a developmental perspective as observed in eye movement patterns. Of special interest in the present study is the question of Semitic word length. Hebrew words, because they are primarily consonantal, tend to be shorter than English words and have less orthographic redundancy (Velan and Frost, 2009). Compared to English words which tend to be morphologically simple (*game*, *play*), Hebrew words are morphologically complex typically consisting of two (or more) interwoven morphemes – the root and pattern (miSXaK “*game*,” leSaXeK “to *play*”).^1^

The present study set out to explore universal and (potentially) language-specific aspects of visual word reading in a non-European language written in a non-alphabetic script. Our study was guided by two main questions. First, we asked if the effects of word familiarity and word length observed in prior research in European languages will also be found in Hebrew, as reflected by eye movement patterns among both university students and elementary-school children. Second, we sought to shed light on developmental differences in eye movement patterns between children and adults. To address these questions, we tracked the eye-movements of skilled adult readers and elementary-school children in Grades 4 to 6 while they read familiar real words and totally unfamiliar words (pseudowords) in Hebrew. The first question was investigated in two ways: differences related to reader’s characteristics were compared by looking at multiple eye movement measures for each of the two age groups, and differences related to word characteristics by comparing two-word types (familiar real words vs. pseudowords). We hypothesized that eye movements of children will reflect longer fixation durations and a higher fixation count than adults. We also hypothesize that pseudowords will elicit longer fixation durations, reflecting greater reliance on the sub-lexical processes.

To answer the second question, we tested the hypothesis that adults and children differ in their eye movement behavior. We predicted fewer fixations per word and shorter fixation durations for adults. Furthermore, we hypothesized an interaction between age and word familiarity and word length. We expect a stronger word familiarity effect among adults than children owing to their greater reliance on parallel whole-word processing. As for the word length effect, to the extent that this effect is universal across writing systems, we predicted a larger word length effect in children than in adults owing to their greater reliance on serial letter-by-letter processing. However, if this effect is modulated by the specific language and writing system factors discussed above, then we might expect to see different patterns to those observed in European languages.

## Materials and methods

### Participants: adults

The participants were from Shechter and Share’s (2021) pupillometry study and included 34 undergraduates from the University of Haifa (age: M = 27.5 years, SD = 5.85, 24 females). All participants were native Hebrew speakers with no learning disabilities or attention deficits and who had normal or corrected-to-normal vision. Four participants were excluded because they did not provide a minimum of 20 valid responses in each of the four conditions (i.e., at least 50% correct responses with no more than 20% missing pupil data). Shechter and Share (2021) only analyzed the pupillary data in their report: the present study analyses the eye movement data. The study was approved by The Ethics Committee of the Department of Education at the University of Haifa (study approval number 18/427). Adult participants voluntarily registered for the experiment and signed an informed consent form prior to the experiment and were compensated in the form of course credit or cash (40 shekels, approximately $12).

### Participants: children

The sample included 38 children from fourth to sixth grades (age: M = 10.4  years, SD = 1.02, 17 females). The four children who reported attentional difficulties were excluded, the remaining participants had no past or present reading difficulties or attentional deficits. In addition, four participants were excluded because they failed to score at least 50% correct responses with no more than 20% missing pupil data. The final sample contained 30 participants, of these participants, nine were fourth graders, 10 were fifth graders, and 11 were sixth graders. Each student and his or her parent signed a voluntary informed consent form prior to the experiment and received a small gift for participation.

### Materials and design

The experiment was a fully within-subjects 2 × 2 design with two levels of familiarity (unfamiliar letter strings [pseudowords] vs. familiar real words) and two lengths (three letters vs. five letters). A total of 160 target stimuli were presented with each of the four conditions containing 40 random items. Additional fillers representing a variety of parts of speech and length were included to provide a more ecologically valid reading condition, 80 items for the adult experiment and 40 for the children. The items were divided into four blocks, each block contained 20 pseudowords (10 of each length), 20 real words (10 of each length) and 20 fillers for adults and 10 fillers for children. All words were of high frequency. For further details concerning the Materials, Design, Stimuli, Procedure and Apparatus see Shechter and Share (2021).

### Apparatus

The eye-movement data were recorded with an EyeLink 1000 Plus (SR Research, Ontario, Canada) with a sampling rate of 1,000 Hz. All experimental materials were presented by using the EyeLink’s Experiment Builder software. A chin rest and head restraint minimized head movements, and participants wore headphones (HS-11 V stereo headphones with SilverLine microphone). The items were presented on a 24-inch LCD monitor (XL24II monitor, BenQ, Taipei, Taiwan; Quadro K620 graphics card, NVIDIA, Santa Clara, CA) with 1,024- × 768-pixel resolution and a refresh rate of 60 Hz. The threshold level for the voice key was defined as 0.1 audio level.

### Procedure

Participants completed the experiment individually in one session that lasted about 30 min. Participants were instructed to read aloud all letter strings (words, pseudowords, and fillers) presented on the screen. At the beginning of each block an instruction screen appeared, then the participant was asked to read aloud the word appearing on the screen. Two practice trials were then presented. A nine-dot calibration was conducted and validated and then the block began. In each trial a fixation cross was presented for 500 ms, followed by a gray fixation screen appearing for 1,000 ms, followed immediately by the stimulus word. The stimuli remained on the screen for 3,300 ms for the adults and 4,700 ms for the children, then the trial ended with a blank screen displayed for 1,500 ms. Pronunciation onset latencies were recorded by a voice key and errors were manually documented by the experimenter.

## Results

Three eye-movement measures were analyzed: first fixation duration, which is the first fixation on the word, dwell time fixation duration which is the sum of all fixation durations made on a word and the number of fixations on a word—fixation count. The time window of interest selected was from item display onset to voice key onset. Fixations shorter than 80 ms were excluded. Only trials with correct responses were included in the analysis; accuracy for adults was 93.4% with 3,094/3,312 trials remaining after data cleaning. Accuracy for children was 84% with 2,659/3,915 trials remained after data cleaning. For each eye-movement measure we removed data points more than ∓ 3 standard deviations from the mean of each group for that measure; total data loss was less than 3%.

Data were analyzed with linear mixed models by using the lme4 package for R (R Core Team, 2021, version 4.1.2). We first ran the analyses for each group separately and specified two fixed factors: word familiarity (categorical: pseudoword and real words) and word length (categorical: three and five letters) and two random effects: subjects and items for all eye movement measures. Furthermore, all fixation duration variables were log transformed prior to analysis (for the descriptive statistics in ms see Table 1). To analyze fixation count we used generalized linear mixed-effects, and due to convergence problems, we retained only subjects as a random variable. All results described here were derived from models that converged successfully. We then analyzed all eye movement measures with both groups combined in the same database by adding the age group as a third fixed factor in the original model. For all of the analyses, we compared the model with no interactions with models with two-way and three-way interactions (in the combined analysis) and arrived at the best possible fit.

**Table 1:** Means and standard deviations (in parentheses) of eye-movement measures (in ms) for two age groups (children and adults), two word types (real and pseudoword), and two word lengths (3 and 5 letters).

		Children			Adults		
Measures		Three letters	Five letters	All words	Three letters	Five letters	All words
First fixation duration	Pseudowords	305 (154)	272 (139)	288 (147)	262 (124)	252 (113)	257 (118)
	Real words	294 (152)	271 (147)	283 (150)	254 (116)	286 (127)	272 (123)
	All word types	300 (153)	272 (143)		258 (120)	271 (121)	
Dwell time duration	Pseudowords	995 (461)	1,090 (501)	1,042 (484)	690 (234)	836 (334)	761 (337)
	Real words	812 (393)	809 (370)	811 (383)	535 (249)	589 (225)	566 (237)
	All word types	898 (436)	949 (462)		613 (299)	696 (301)	
Fixation count	Pseudowords	4.2 (1.6)	4.9 (1.7)	4.6 (1.7)	2.6 (1.2)	3.3 (1.3)	3.0 (1.3)
	Real words	3.5 (1.4)	3.8 (1.5)	3.6 (1.5)	2.1 (1.0)	2.4 (1.0)	2.3 (1.0)
	All word types	3.9 (1.6)	4.4 (1.7)		2.4 (1.1)	2.8 (1.3)	

**Figure 1:**
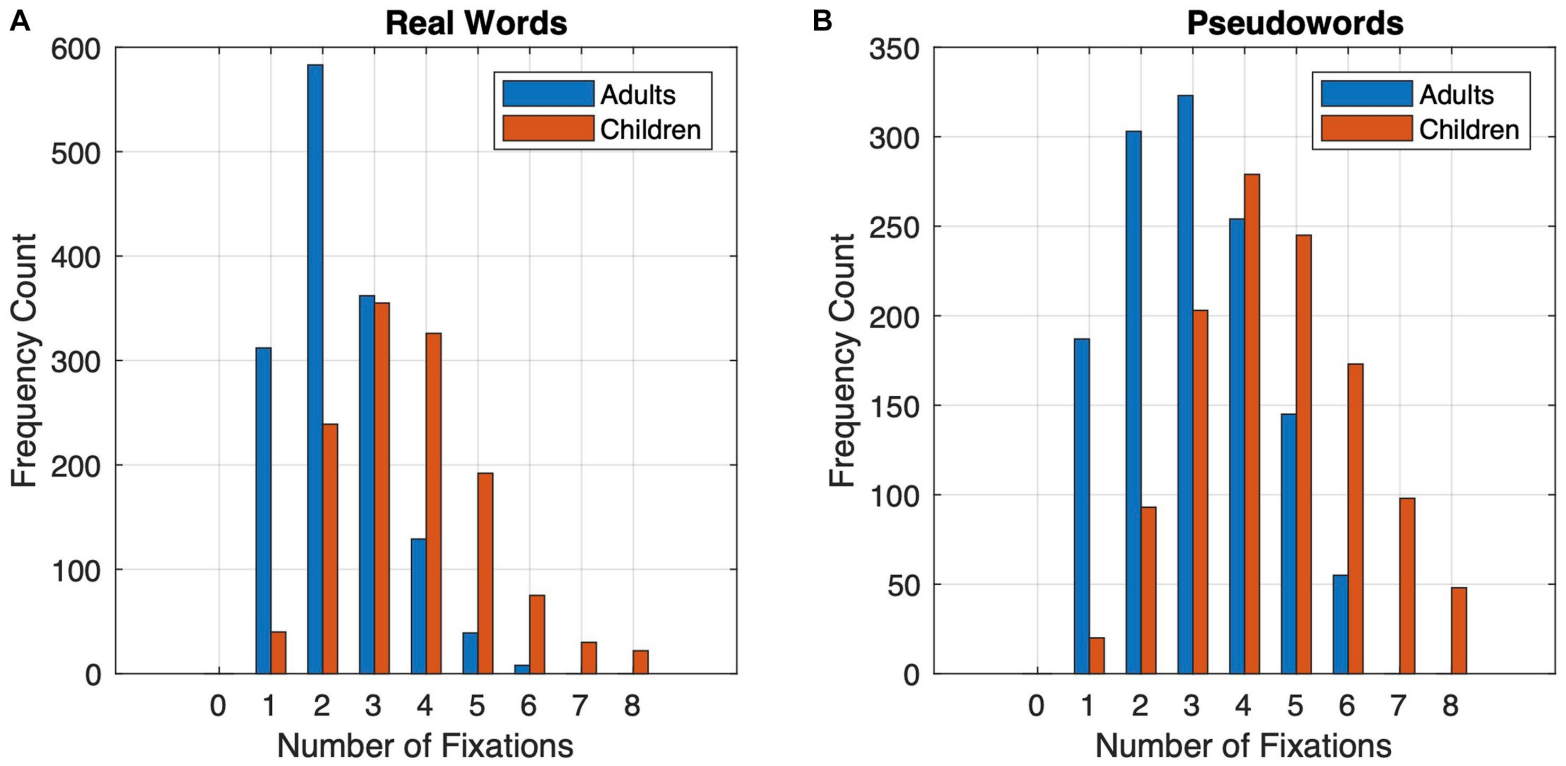
Number of fixations made on words during reading **(A)** real words and **(B)** pseudowords for adults and children.

Our first hypothesis predicted that the eye movements of children will reflect a lengthier reading process. The data supported this hypothesis: first, as seen in Table 1, the global eye movement measures of children are consistent with the hypothesis of a slower, more sequential letter-by-letter reading process as seen in the dwell time fixation durations and in the number of fixations made on words. Second, and as predicted by the word familiarity hypothesis, pseudowords were associated with longer dwell time fixation durations and a higher fixation count. This is also seen in Figure 1 which shows that pseudowords resulted in a higher frequency of three or more fixations (Figure 1B) than real words where the frequency was highest on two and three fixations (Figure 1A). In addition, the figure shows the difference in the distribution of number of fixations between children and adults. We return to these differences in the analyses of the combined data.

### Children’s eye-movements

The LME summary statistics are presented in Table 2. Recall we had two fixed factors—word length and word familiarity. The model with the two-way interaction was the best fit.

**Table 2:** LME model summary statistics for eye-movement measures in children.

Fixed effects	First fixation duration			Dwell time duration			Fixation count		
	β	SE	t	β	SE	t	β	SE	z
Intercept	5.52	0.04	122.3^***^	6.74	0.05	114.18^***^	1.43	0.04	32.12^***^
Word type	−0.02	0.03	−0.75	−0.20	0.03	−5.42^***^	−0.18	0.02	−6.68^***^
Word length	−0.09	0.03	−2.62^*^	0.12	0.03	3.34^**^	0.16	0.02	6.20^***^
Word type × word length interaction	–	–	–	−0.11	0.05	−2.09^*^	−0.08	0.03	−2.11^*^

^+^*p* < 0.10, ^*^p < 0.05, ^**^p < 0.01, ^***^p < 0.001.

#### Word familiarity

The effect of word familiarity was significant in the dwell time fixation duration measure and fixation count, but not in first fixation duration. Pseudowords were associated with longer dwell time fixation duration (M = 1,047; SE = 44.3) than real words (M = 800; SE = 44.3) and had a higher fixation count (M = 1.51; SE = 0.04) than real words (M = 1.28; SE = 0.04).

#### Word length

The effect of word length was significant in all three eye movement measures. In first fixation duration, contrary to the standard word length effect in English, five letter words were associated with *shorter* first fixation duration (M = 274; SE = 10.1) than three letter words (M = 303; SE = 10.1). However, in the total dwell time the reverse was true, five letter words were associated with longer dwell time fixation duration (M = 955; SE = 44.3) than three letter words (M = 892; SE = 44.2). In addition, longer words had a significantly higher fixation count (M = 1.46; SE = 0.04) than short words (M = 1.34; SE = 0.04).

#### Word length by word familiarity interaction

**Figure 2:**
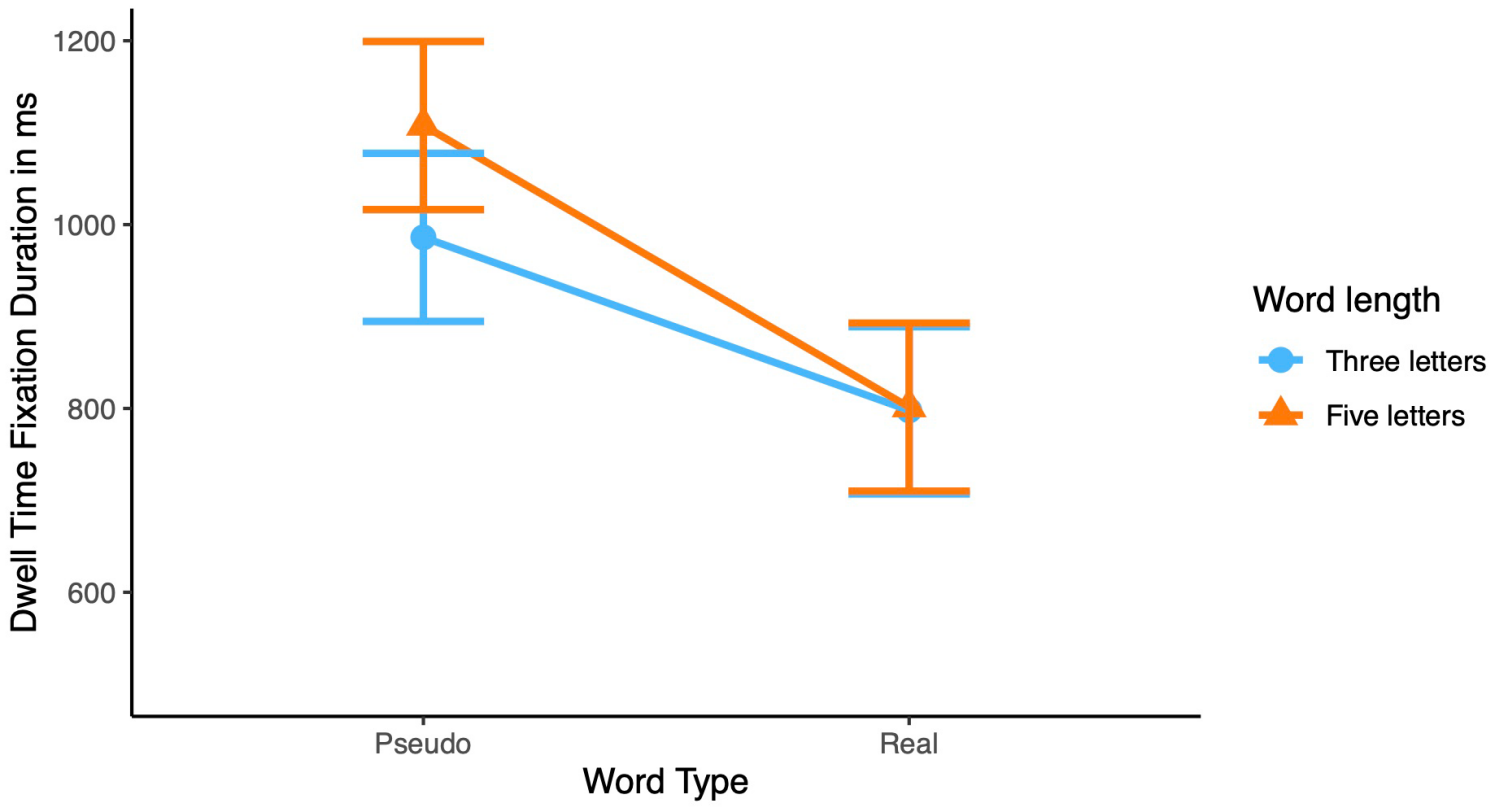
Interaction of word type and word length on dwell time fixation duration in children.

**Figure 3:**
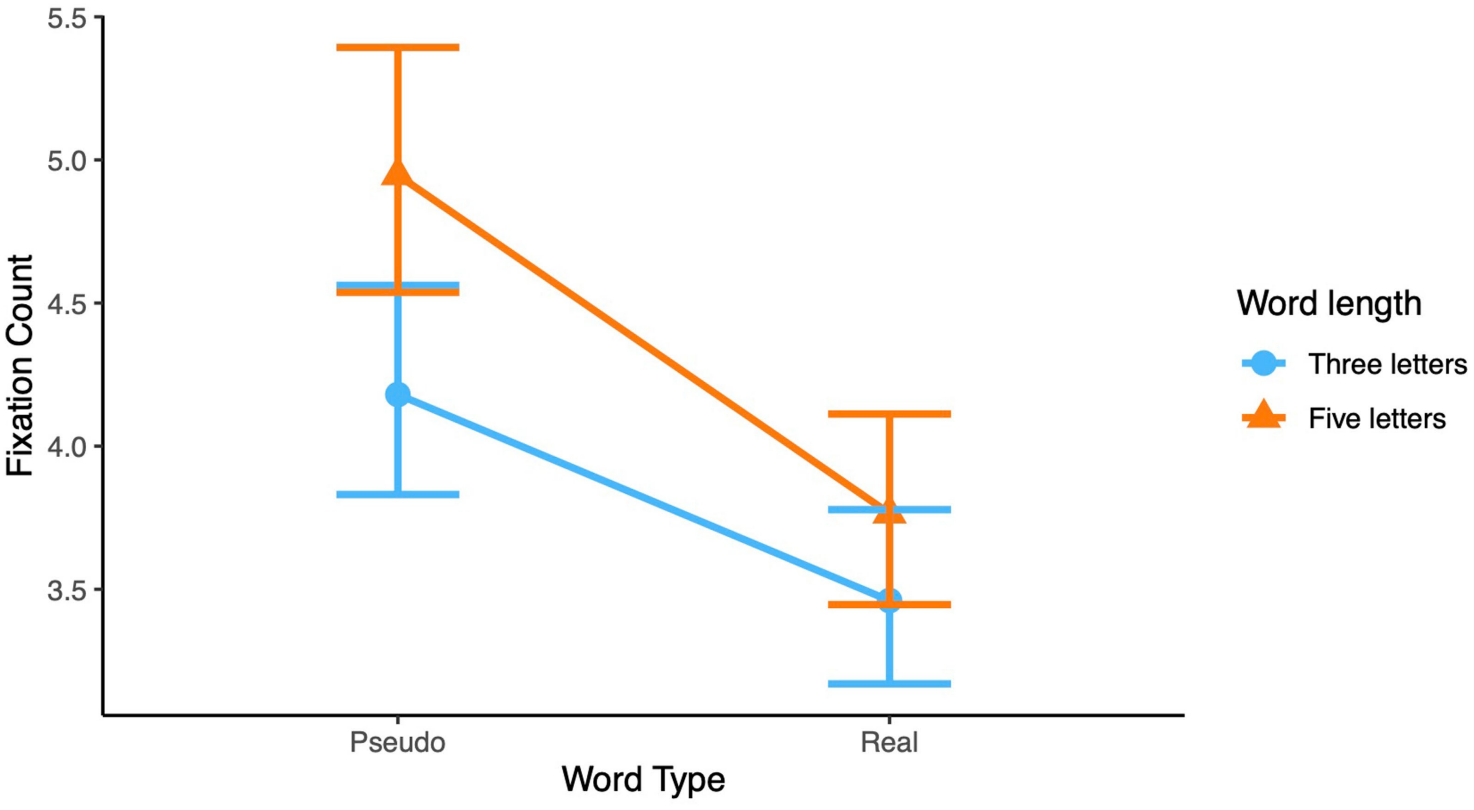
Interaction between word type and word length on fixation counts for children.

This interaction was significant in dwell time fixation duration and in fixation count. In dwell time, this interaction revealed that for real words no difference was observed in dwell time duration between three letters and five letter words. Pseudowords, however, resulted in significantly longer dwell time durations for longer words than for shorter words, see Figure 2. For fixation count this interaction also revealed the standard finding that the difference between three letters and five-letters words for pseudowords was larger than for real words, see Figure 3.

To summarize, the word familiarity effect was evident throughout the entire reading processes of children, showing longer reading processes for pseudowords. This effect interacted with word length in dwell time fixation duration consistent with the idea of holistic reading of real words (hence the absence of a word length effect), in contrast to serial processing of pseudowords generating longer dwell times on longer words than on shorter words. We return to the counterintuitive first fixation finding for word length in the Discussion.

### Adults’ eye-movements

The LME summary statistics are presented in Table 3. The model with no familiarity by word length interaction emerged as the best fit.

**Table 3:** LME model summary statistics for eye-movement measures in adults.

Fixed effects	First fixation duration			Dwell time duration			Fixation count		
	β	SE	t	β	SE	t	β	SE	z
Intercept	5.40	0.04	124.08^***^	6.37	0.05	122.23^***^	1.20	0.05	21.38^***^
Word type	−0.01	0.03	−0.58	−0.30	0.02	−11.08^***^	−0.26	0.02	−10.74^***^
Word length	−0.02	0.03	−0.68	0.18	0.02	6.89^***^	0.21	0.02	8.90^***^

^+^*p* < 0.10, ^*^p < 0.05, ^**^p < 0.01, ^***^p < 0.001.

#### Word familiarity

The effect of word type was significant in dwell time fixation duration and in fixation count but not in first fixation duration. Pseudowords were associated with significantly and substantially longer dwell time durations (M = 751; SE = 25.7) than real words (M = 548; SE = 25.6). In addition, pseudowords were associated with a higher fixation count (M = 1.05; SE = 0.04) than real words (M = 0.79; SE = 0.04).

#### Word length

The effect of word length was significant in dwell time fixation duration and fixation count, but, once again, not in first fixation duration. Five letter words elicited longer dwell time fixation durations (M = 704; SE = 25.6) than three letter words (M = 595; SE = 25.7) and had a higher fixation count (M = 1.03; SE = 0.04) than did three letter words (M = 0.81; SE = 0.04). Taken together, the results from the adult sample are similar to the children’s data and emphasize that pseudowords and longer words take more time to process.

### Developmental analysis: adults vs. children

The developmental analysis was carried out on the data from both samples and directly compared the two age groups. Analyzing the same eye movement measures, we now had three fixed factors: age group, word familiarity and word length. The model with two-way interactions was the best fit. The LME model summary statistics are presented in Table 4.

**Table 4:** LME model summary statistics for eye-movement measures in the developmental analysis.

Fixed effects	First fixation duration			Dwell time duration			Fixation count		
	β	SE	t	β	SE	t	β	SE	Z
Intercept	5.55	0.04	133.60^***^	6.77	0.04	146.77^***^	1.45	0.03	41.04^***^
Age group	−0.14	0.04	−3.52^***^	−0.42	0.03	−13.43^***^	−0.52	0.02	−18.18^***^
Word type	−0.04	0.04	−1.13	−0.20	0.03	−6.44^***^	−0.18	0.02	−7.23^***^
Word length	−0.11	0.04	−2.73^**^	0.13	0.03	4.15^***^	0.17	0.02	6.96^***^
Age group × word type interaction	0.01	0.04	0.37	−0.03	0.03	−1.00	−0.02	0.03	−0.75
Age group × word length interaction	0.08	0.04	1.84+	0.12	0.03	3.52^***^	−0.08	0.03	2.77^**^
Word type × word length interaction	–	–	–	−0.12	0.03	−3.40^***^	−0.09	0.03	−2.91^**^

^+^*p* < 0.10, ^*^p < 0.05, ^**^p < 0.01, ^***^p < 0.001.

#### Age effect

As predicted, age significantly affected eye movements. Overall, children’s reading was associated with longer dwell time fixation duration than adult’s reading and had a significantly higher fixation count and longer first fixation durations.

#### Word familiarity

The effect of word familiarity was observed in dwell time fixation duration where real words resulted in shorter dwell time fixation duration (M = 678; SE = 28.3) than pseudowords (M = 905; SE = 28.3). Moreover, the fixation count on pseudowords was significantly higher (M = 1.30; SE = 0.03) than on real words (M = 1.06; SE = 0.03).

#### Word length

The overall effect of word length was observed in all three measures. Three letter words, once again, and contrary to the standard finding in English, had significantly higher first fixation durations (M = 286; SE = 7.4) than five letter words (M = 266; SE = 7.37), reflecting the effect observed in the children’s analyses. However, when considering the entire process as observed in fixation count and dwell time fixation duration, the reverse is found. Five letter words generated significantly longer dwell time fixation durations (M = 834; SE = 28.3) than three letter words (M = 748; SE = 28.3) and had higher fixation count (M = 1.26; SE = 0.03) than three letter words (M = 1.09; SE = 0.03).

#### Age group by word familiarity interaction

We predicted a stronger word familiarity effect among adults than children, however this interaction was not significant in any of the tested eye movement measures, indicating that both adults and children in Grades 4–6 show similar familiarity effects.

#### Age group by word length interaction

**Figure 4:**
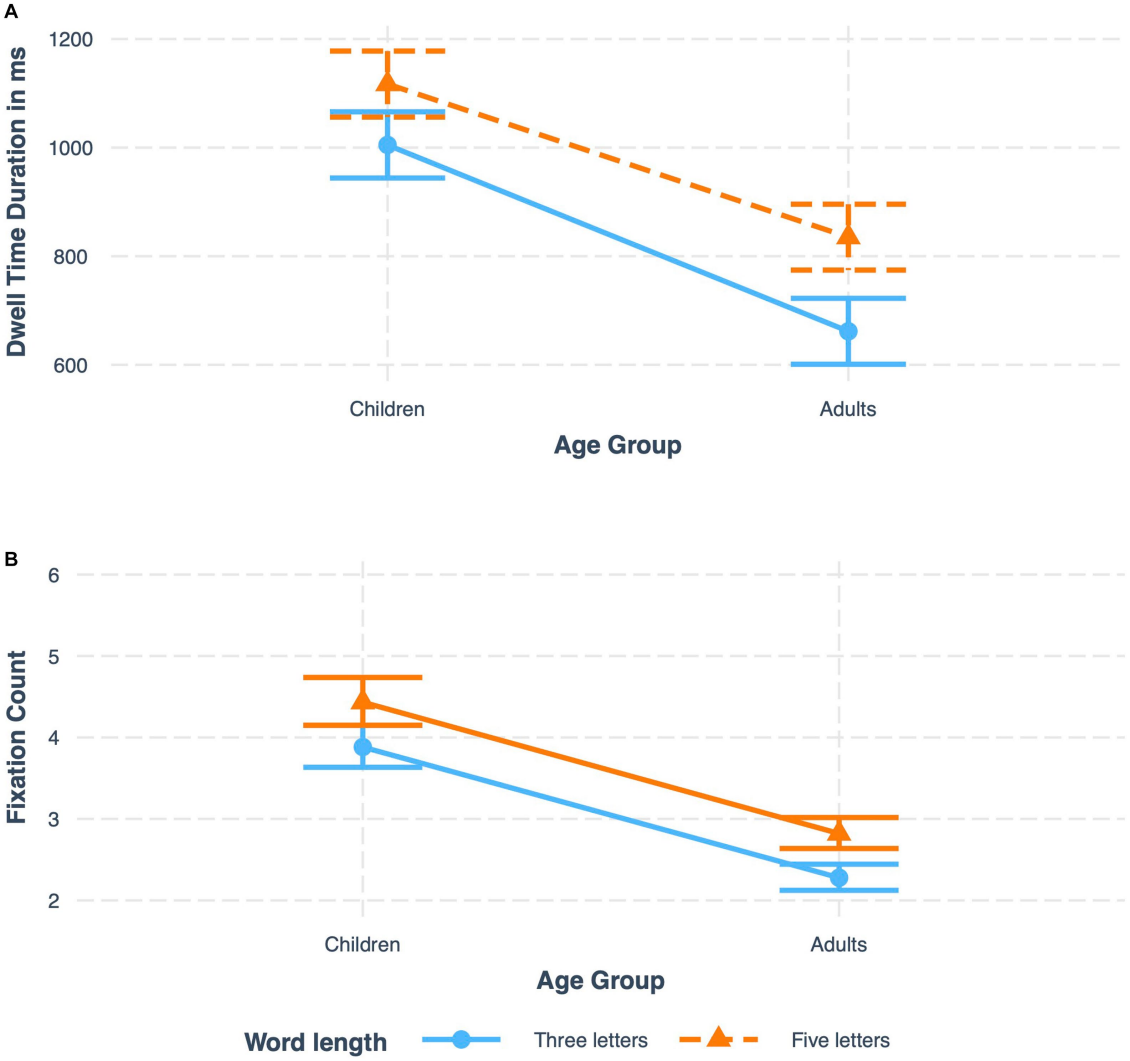
Interaction of age group and word length on **(A)** dwell time fixation duration and **(B)** fixation count.

**Figure 5:**
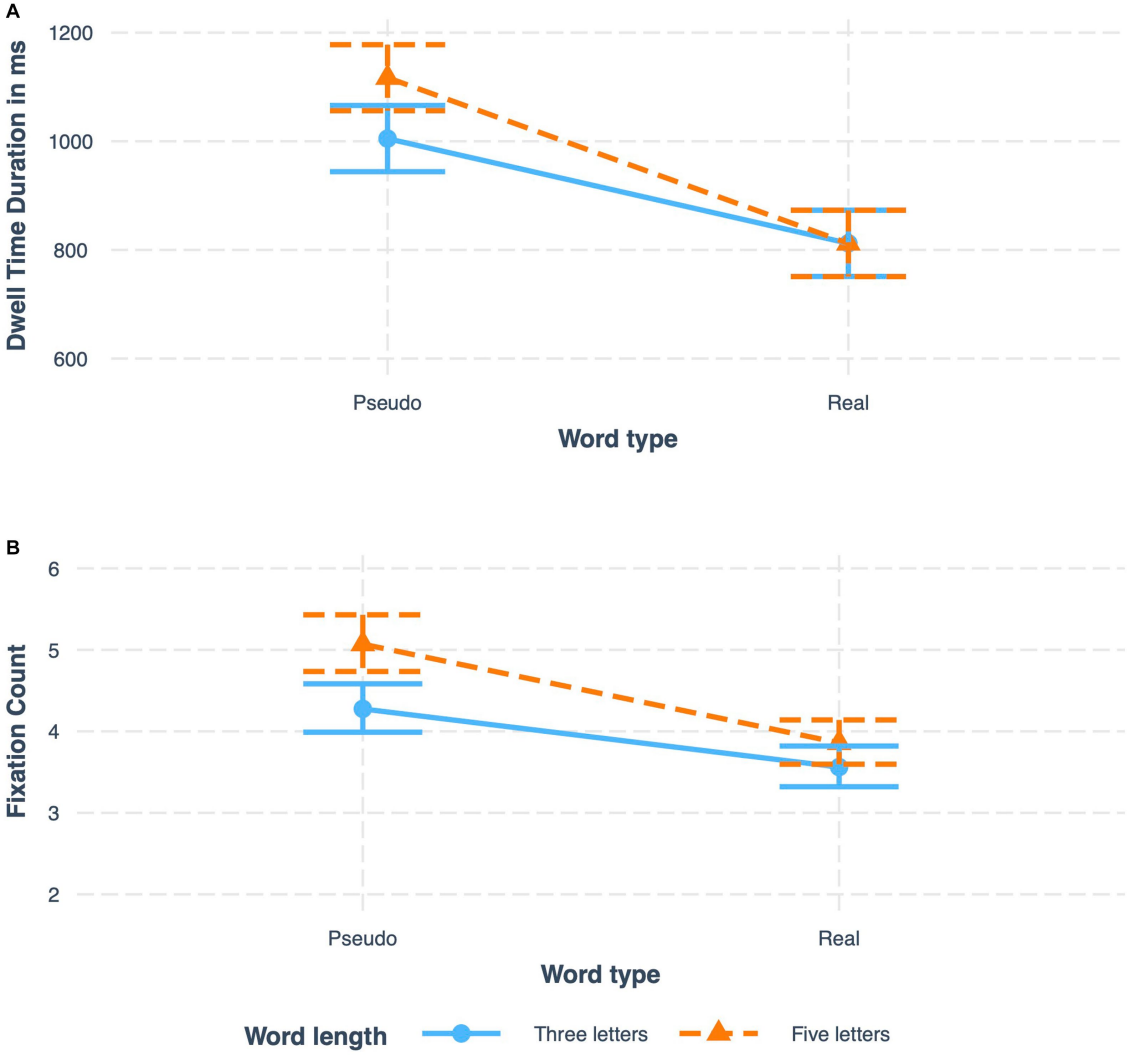
Interaction of word familiarity and word length on **(A)** dwell time fixation duration and **(B)** fixation count.

We predicted an interaction between word length and age group, anticipating a stronger effect for word length in children than in adults. This interaction was significant in dwell time fixation duration and fixation count. Results revealed that in both children and adults, five letter words were associated with longer dwell time durations, however, contrary to expectations, the difference in dwell time between five letter and three letter items was *larger* for adults (117 ms) than for children (56 ms), see Figure 4 (upper panel). In addition, this interaction was significant in the fixation count measure where the difference between the number of fixations on three letters and five-letter words is slightly larger among adults, see Figure 5 (lower panel).

#### Word familiarity by word length interaction

Although this interaction was not part of the hypothesis, results revealed that in dwell time duration and fixation count this interaction was significant indicating a larger length effect for pseudowords than for real words, similar to the interaction which was found in the children’s analyses (see Figure 5).

## Discussion

This study set out to explore universal and language-specific aspects of visual word reading in Hebrew—a non-European language written in a non-alphabetic script. Most reading models have been derived from European languages, such as the dual route (Coltheart et al., 1993; Zorzi, 2010; Ellis and Young, 2013), and the applicability of such models to different writing systems remains unclear. Against the theoretical backdrop of the unfamiliar-to-familiar dualism proposed by Share (2008), we asked whether differences between children and adults would reflect a shift from the sub-lexical route to the lexical route. We also directly examined the familiarity effect and the word length effect in Hebrew, again adopting a developmental perspective.

Our results revealed two main findings relating to universal (i.e., cross-linguistic/cross-script) aspects of word reading. First, the familiarity effect observed in adults and in children confirmed that reading unfamiliar letter strings (pseudowords) entails a longer process than reading familiar real words as seen in longer fixation durations and number of fixations on words. This finding is comparable to previous findings (e.g., De Luca et al., 2002; Hautala et al., 2011; Wochna and Juhasz, 2013) and aligns well with both the classic Coltheart/Baron dual route model and Share’s more universal unfamiliar-to-familiar dualism. The familiarity finding is typically interpreted as reflecting the involvement of the faster whole-word or lexical route in reading real words and the slower sequential (sub-lexical) route in reading pseudowords (and unfamiliar words more generally) which is characterized by longer fixations and a higher fixation count. This interpretation, of course, is a post-hoc account which calls for more direct evidence which we are currently pursuing in our lab. The second finding revealed that children’s word reading overall points to a more time-consuming and/or less efficient process compared to adults (see Shechter et al., 2022) as observed in the longer fixation durations and the larger number of fixations.

Regarding script-specific aspects of word reading, we also set out to examine the word length effect and explore how it might be modulated by the specific features of the language and writing system. As noted in numerous studies in European alphabetic writing systems (Weekes, 1997; Ans et al., 1998; Rayner, 1998; Wang et al., 2019), the word length effect was evident in our study in both the children’s and the adults’ analyses; readers recognized shorter words rapidly, whereas longer words needed additional fixations and dwell time fixation durations.

However, when considering the combined data analyses, the interaction between word factors and age group did not follow the conventional pattern, namely, a stronger word length effect among children compared to adults due to a more serial letter-by-letter processing and a stronger word familiarity effect among adults reflecting more parallel whole-word processing in word recognition. Contrary to expectations, this study did not find a significant interaction between word familiarity and age group. This might be due to the fact that the younger participants were already fluent readers and therefore showed a similar reading pattern to adults in reading pseudowords and real words. According to the Triplex model of Hebrew reading acquisition (Share and Bar-On, 2018), the transition from primarily bottom-up letter by letter decoding to whole-word, morpho-lexical-orthographic word identification typically occurs in Grade 2, whereas our children were from Grades 4, 5, and 6.

The interaction of word length with age group revealed a surprising pattern – the effect of word length was *larger* for adults than children in both dwell time fixation duration and fixation count. Previous studies in European languages showed a decreasing word length effect in adults as compared to children and dysfluent readers (De Luca et al., 2002; Spinelli et al., 2005; Zoccolotti et al., 2005; Martens and de Jong, 2006, 2008). However, we found that length was *more* conspicuous in adults’ reading than children implying that word length influences eye movements” in Hebrew is a different way than it does in European languages. While more research is needed to replicate this finding, it may be related to the unique properties of Semitic morphology and what Frost (1995) termed “degrees of freedom.” This is the idea that, in *unpointed* Hebrew (which is only partially vowelled by means of the non-optional vowel letters), the diacritic-like vowel signs are dropped (around Grade 3), leaving the reader to fill in the “gaps” and supply the missing vowel information by themselves on the basis of lexical and morphological knowledge. However, for some words, the number of possible and permissible vowel patterns (“degrees of freedom”) is large, whereas in others it is small. We suspect that our three-letter words actually had *more* degrees of freedom (or ambiguity) regarding vowel patterns because far more morphological patterns exist for these shorter words (which contain only root letters) than for the longer five-letter words which in our study included many affixed consonants that specify the morpho-phonological pattern and hence the specific vowelling pattern. Although in our study, all words were fully pointed (i.e., all vowels (as well as consonants) are unambiguously marked), it is well known that after Grade 1, when children are taught all the consonantal letters and vowel signs, knowledge of the vowel signs declines rapidly (see, for example, Shany et al., 2012) and is replaced by reliance on higher-order information such as morphological patterns and specific lexical knowledge (Share, 2017). With regard to the age by word length interaction, we speculate that although this effect operates on both age groups, it is stronger in adults compared to children. This is because adults are less exposed to pointed script and also have greater lexical knowledge and hence are more aware of the many possible alternative pronunciations of the letter strings. This finding raises fundamental questions regarding cross-linguistic differences in length effects (at least between European alphabets and Semitic abjads). Future research should consider including a direct comparison between Hebrew and English.

In conclusion, our data highlight both universal and unique aspects of visual word recognition. With regard to language-specific effects, it appears that word length may be uniquely influenced by morphology and pointing which is unique to Semitic languages such as Hebrew and Arabic. With regard to universal effects, our developmental data confirmed the well-established findings regarding longer dwell times and higher fixation counts among younger less skilled readers. Another universal aspect of our data relates to word familiarity, namely the strong and reliable advantage for familiar words compared to unfamiliar words which has been shown to be a cross-linguistic universal (Share, 2008; Verhoeven and Perfetti, 2022).

## Data availability statement

The raw data supporting the conclusions of this article will be made available by the authors, without undue reservation.

## Ethics statement

Ethical approval for all the experiments was granted by the Ethical Committee of the Faculty of Education of the University of Haifa (No. 18/427). Written informed consent to participate in this study was provided by the participants’ legal guardian/next of kin.

## Author contributions

All authors listed have made a substantial, direct, and substantive contribution to the work and approved it for publication.

## Funding

This research was supported by the Ministry of Science and Technology, Israel; Israel Science Foundation (grant no. 1002/20 to DS); and the Edmond J. Safra Brain Research Center for the Study of Learning Disabilities.

## Conflict of interest

The authors declare that the research was conducted in the absence of any commercial or financial relationships that could be construed as a potential conflict of interest.

## Publisher’s note

All claims expressed in this article are solely those of the authors and do not necessarily represent those of their affiliated organizations, or those of the publisher, the editors and the reviewers. Any product that may be evaluated in this article, or claim that may be made by its manufacturer, is not guaranteed or endorsed by the publisher.

## Footnotes

^1^Root letters (consonants) are capitalized, morphological pattern letters (consonants and vowels) are in lowercase letters.
